# Serial high-resolution ultrasonography demonstrates dynamic inflammatory changes during multidrug therapy in leprosy neuropathy: a longitudinal case report

**DOI:** 10.1186/s12883-026-05152-5

**Published:** 2026-07-11

**Authors:** Hendra Gunawan, Risa Miliawati Nurul Hidayah, Eva Krishna Sutedja, Trustia Rizqandaru, Yulisa Handayani

**Affiliations:** Department of Dermatology and Venereology, Faculty of Medicine, Universitas Padjadjaran - Dr. Hasan Sadikin General Hospital, Bandung, West Java Indonesia

**Keywords:** High-resolution ultrasonography, *Leprosy neuropathy*, *Multi-drug therapy*, *Nerve impairment*

## Abstract

**Background:**

Leprosy is a chronic granulomatous infection caused by *Mycobacterium leprae*, the leading infectious cause of nerve impairment-related disability. Peripheral neuropathy may occur before, during, or after diagnosis and treatment or as a result of leprosy reactions. High-resolution ultrasonography (HRUSG) provides a more precise evaluation of peripheral nerve abnormality in leprosy patients than clinical examination alone. Longitudinal data assessing dynamic alterations following multidrug treatment (MDT) using HRUSG are limited, especially in endemic settings. We report two leprosy patients who underwent HRUSG evaluation before and after MDT.

**Case presentation:**

Case 1: A 20-year-old male with tuberculoid leprosy presenting with a hypoesthetic hypopigmented plaque on the left arm without palpable nerve enlargement. HRUSG performed before and three months after MDT revealed no nerve abnormalities. Case 2: A 47-year-old male with borderline lepromatous leprosy and severe reversal reaction presented with multiple hypoesthetic erythematous plaques, finger shortening, and stocking anesthesia, without palpable nerve thickening. Pre-treatment HRUSG markedly increased cross-sectional area (CSA) (e.g., ulnar nerve up to 37.0 mm²), hypoechogenicity, and hypervascularity. After two months of MDT combined with corticosteroids, CSA decreased across examined nerves (e.g., ulnar nerve to 27.2 mm²), and hypervascularity resolved in several sites, while hypoechogenicity persisted.

**Conclusions:**

This first longitudinal HRUSG comparison of TT and BL leprosy cases in Indonesia demonstrates that serial ultrasonography can detect subclinical nerve involvement and document early structural improvement during therapy. HRUSG may serve as a non-invasive adjunct for monitoring inflammatory activity and therapeutic response in leprosy neuropathy.

## Background

Leprosy, also known as Hansen’s disease, is a slowly progressive, chronic bacterial infection caused by *Mycobacterium leprae* (*M. leprae*) or *Mycobacterium lepromatosis* [1] with 172,711 new cases detected in 188 countries predominantly from India, Brazil, and Indonesia [2]. Indonesia ranks third globally in leprosy incidence, with over 14,698 new cases reported by 2024 [3].

The peripheral nerves are among the systems impacted by *M. leprae*’s invasion. Damage to peripheral nerves can result in sensory and motor impairment, which may lead to deformities and disabilities [1]. Neurological involvement may occur before, during, or after diagnosis and treatment and may also result from leprosy reactions. This can result in functional impairments and deformities that necessitate immediate treatment [4, 5]. 

In order to detect nerve involvement, palpation can identify nerve thickness, although it is dependent on the expertise of the examiner and requires practical training [4, 6]. A recent study has demonstrated that high-resolution ultrasonography (HRUSG) of nerves in leprosy patients reveals a number of notable changes, including increased vascularity, distorted echotexture, and enlargement of nerves. Moreover, compared to clinical observations, HRUSG identified more extensive damage and a greater number of affected nerves [7]. A sonological abnormality was established when at least one of the following parameters was identified: (1) An increased cross-sectional area (CSA); (2) Hypoechogenicity of the nerve (fascicles and interfascicular perineurium), with or without loss of architecture; and (3) Hypervascularity. HRUSG offers an objective, non-invasive tool to assess nerve involvement, but its role in monitoring therapeutic response remains underreported [6]. 

We hypothesized that serial HRUSG would reveal measurable structural and vascular changes during treatment, particularly in patients with inflammatory activity, even in the absence of clinically palpable nerve enlargement. Here, we report two newly diagnosed leprosy cases: tuberculoid leprosy (TT) and borderline lepromatous leprosy (BL) with severe reversal reaction (RR) evaluated by HRUSG before and after MDT. This report represents the first longitudinal HRUSG comparison of TT and BL cases in Indonesia, highlighting its utility in monitoring therapeutic responses to leprosy and RR.

## Case presentation

### Case 1

A 20-year-old male from an endemic area presented with a hypopigmented plaque on the left arm that was not itchy or painful for one year. The initial lesion was a button-sized hypopigmented macule, which gradually enlarged to coin size. The patient self-medicated with Betason N^®^ and ketoconazole cream from a pharmacy, but saw no improvement. The patient reported no previous or present complaints of nerve pain, skin lesion tenderness, or sudden worsening of sensory or motor symptoms. There were no similar complaints from the patient’s family or neighbors. Aside from skin problems, the patient had no additional concerns.

**Fig. 1:**
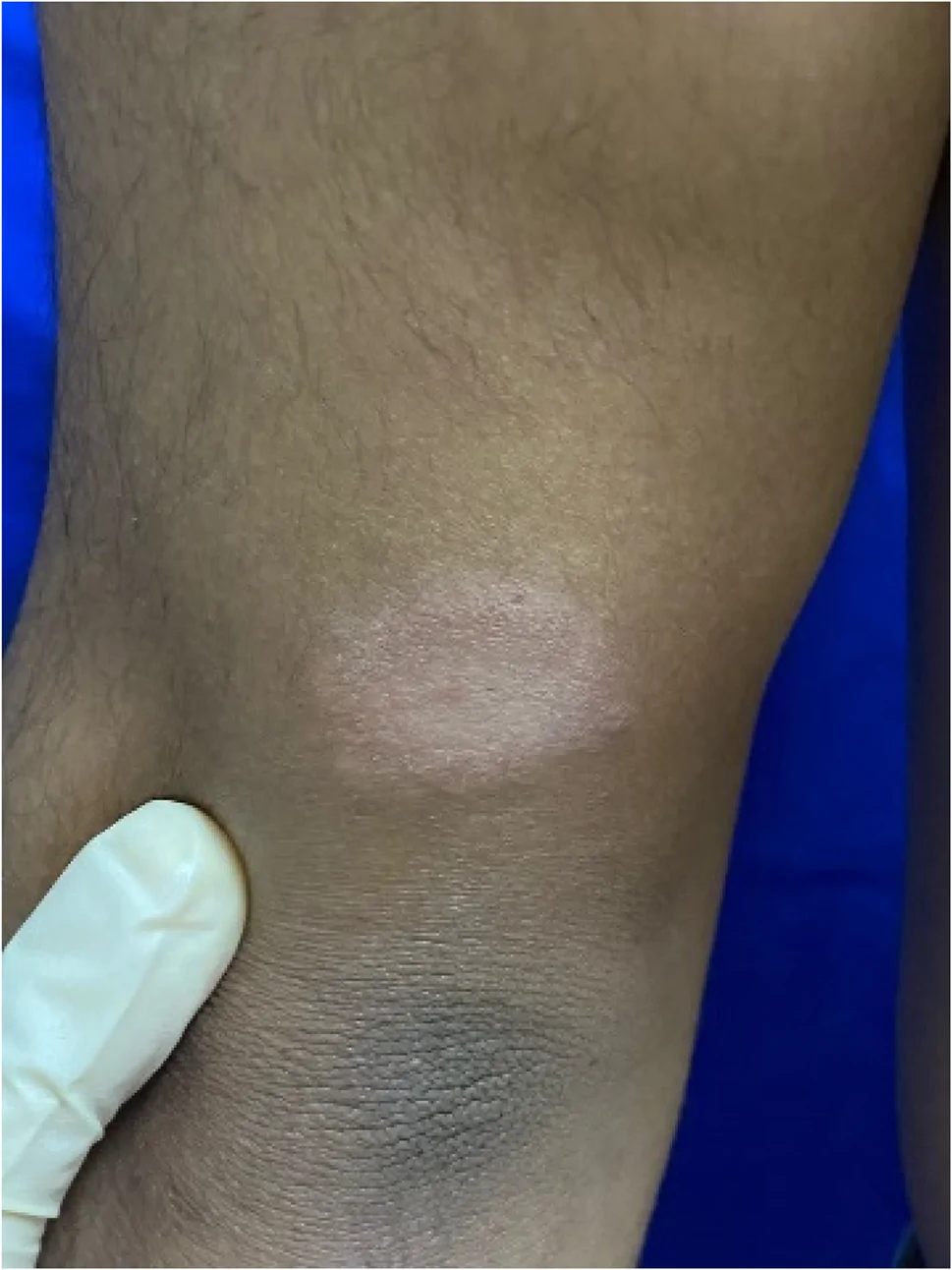
Case 1. Hypoesthetic hypopigmented plaque on the left arm in the TT patient before treatment

**Fig. 2:**
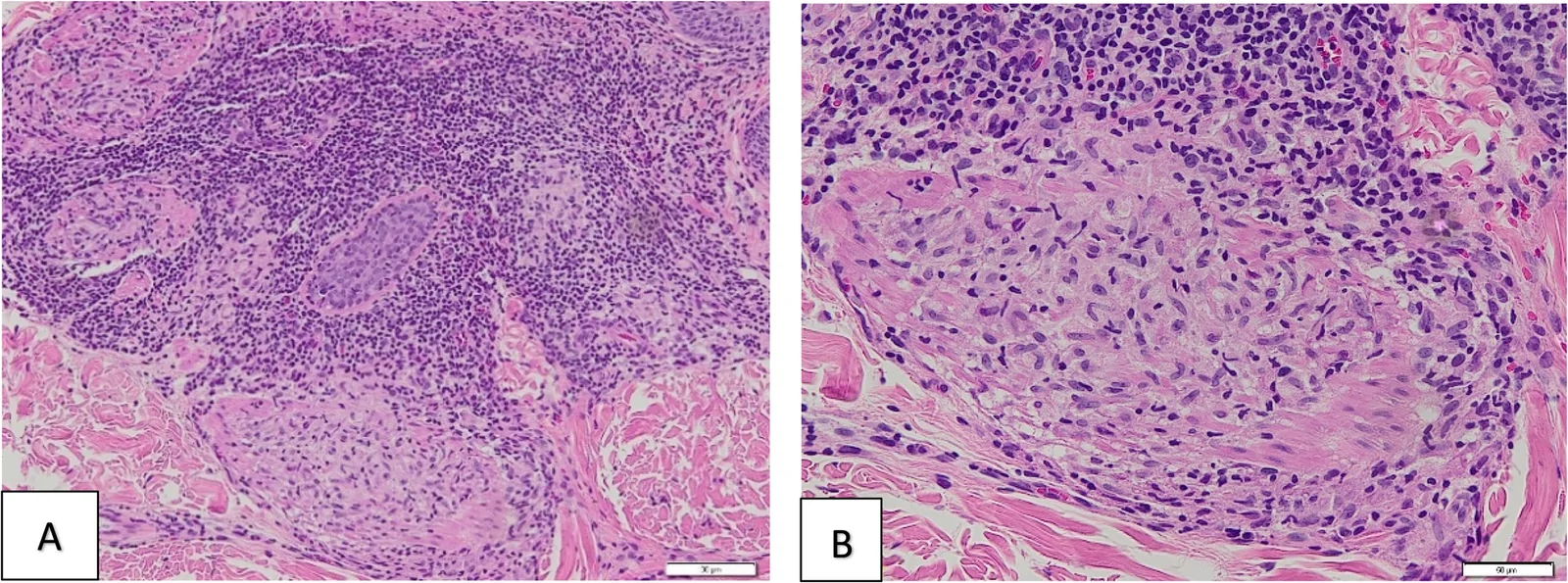
Case 1. The histopathological examination demonstrated a granulomatous inflammation with lymphocyte infiltration (**A**) and epithelioid cells (**B**) supporting the diagnosis of tuberculoid leprosy. (Hematoxylin-Eosin stain; original magnification, 100x (**A**), 200x (**B**), respectively)

**Fig. 3:**
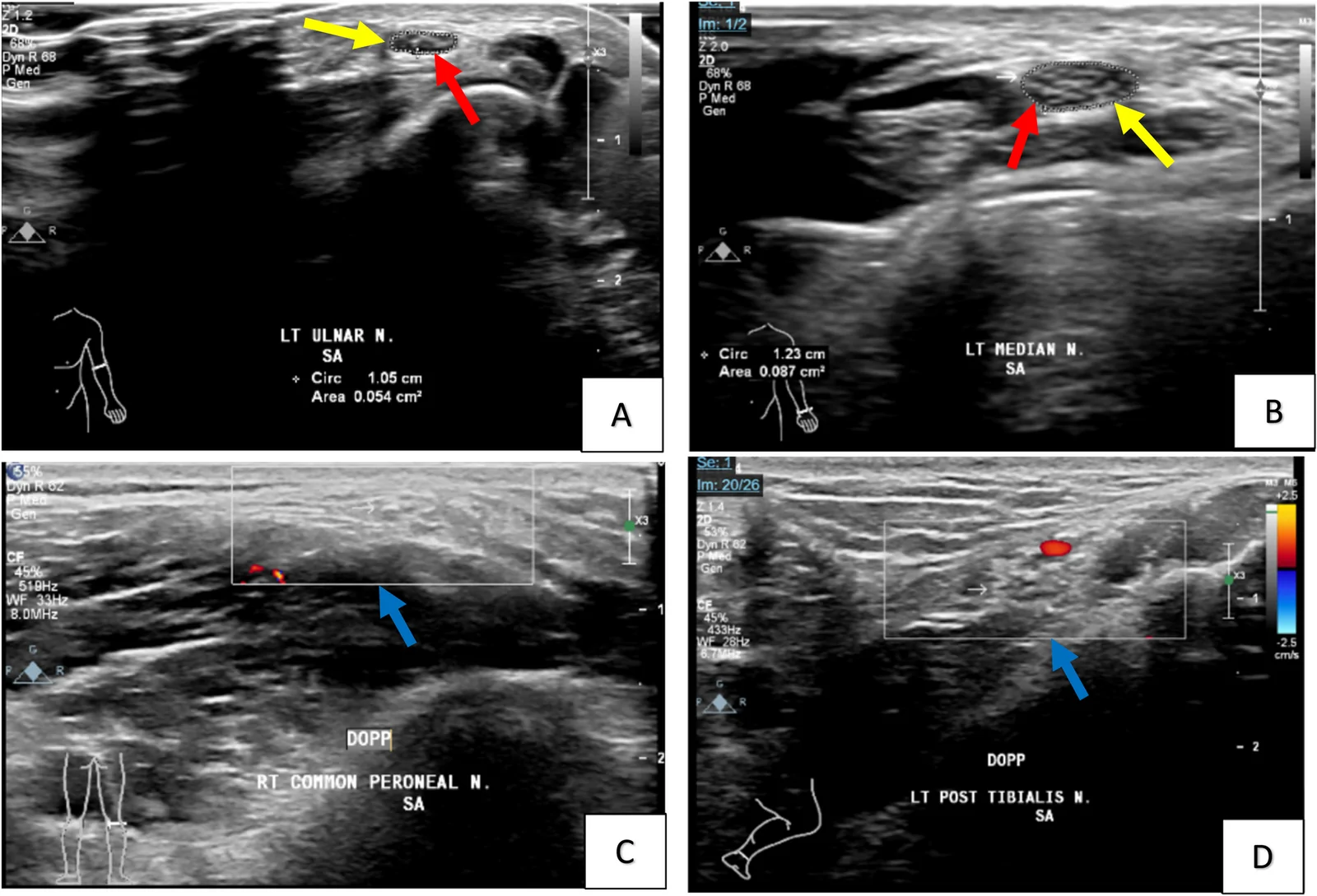
Case 1. Pre-treatment, HRUSG of the ulnar (**A**), median (**B**), common peroneal (**C**), and posterior tibial nerves (**D**) in patients with TT-type leprosy revealed no hypoechogenicity (yellow arrow), no increased CSA (red arrow) and the absence of hypervascularity (blue arrow)

Physical examination revealed a solitary nummular plaque with sensory loss (Fig. 1), but palpation of the lesion and perilesional area detected no nerve enlargement. No motor weakness was found, but motor reflexes were not tested. Slit-skin smear was negative for acid-fast bacilli (AFB). Histopathology showed a granulomatous reaction with epithelioid cells, Langhans giant cells, and lymphocyte infiltration, suggestive of leprosy (Fig. 2A and B). Polymerase chain reaction (PCR) examination was not done. The patient underwent an HRUSG examination using a Philips^®^ EPIQ Elite system with an eL18-4 linear transducer (frequency range: 4–18 MHz) prior to the initiation of MDT. Echogenicity, CSA, and vascularity were evaluated in the ulnar nerve at the cubital tunnel, the median nerve proximal to the carpal tunnel, the common peroneal nerve at the fibular head, and the posterior tibial nerve at the medial malleolus, performed by a single experienced examiner. Vascularity was evaluated qualitatively via Color Doppler (CD), with settings adjusted for the identification of low-flow signals. Increased intraneural vascular signals were interpreted as hypervascularity. Currently, no standardized quantitative criteria or validated grading system exists for classifying hypervascularity in peripheral nerve leprosy; therefore, vascularity assessment was based on qualitative evaluation of Doppler signals. No abnormalities were identified in the peripheral nerves (Fig. 3A − D). These findings established a diagnosis of TT-type leprosy based on a clinical-histological-HRUSG examination. The patient was planned to be treated with MDT- paucibacillary (PB) for six months. After administering MDT for three months, the lesion resolved to a hypopigmented macule, and a repeat HRUSG examination revealed no evidence of nerve abnormality.

### Case 2

**Fig. 4:**
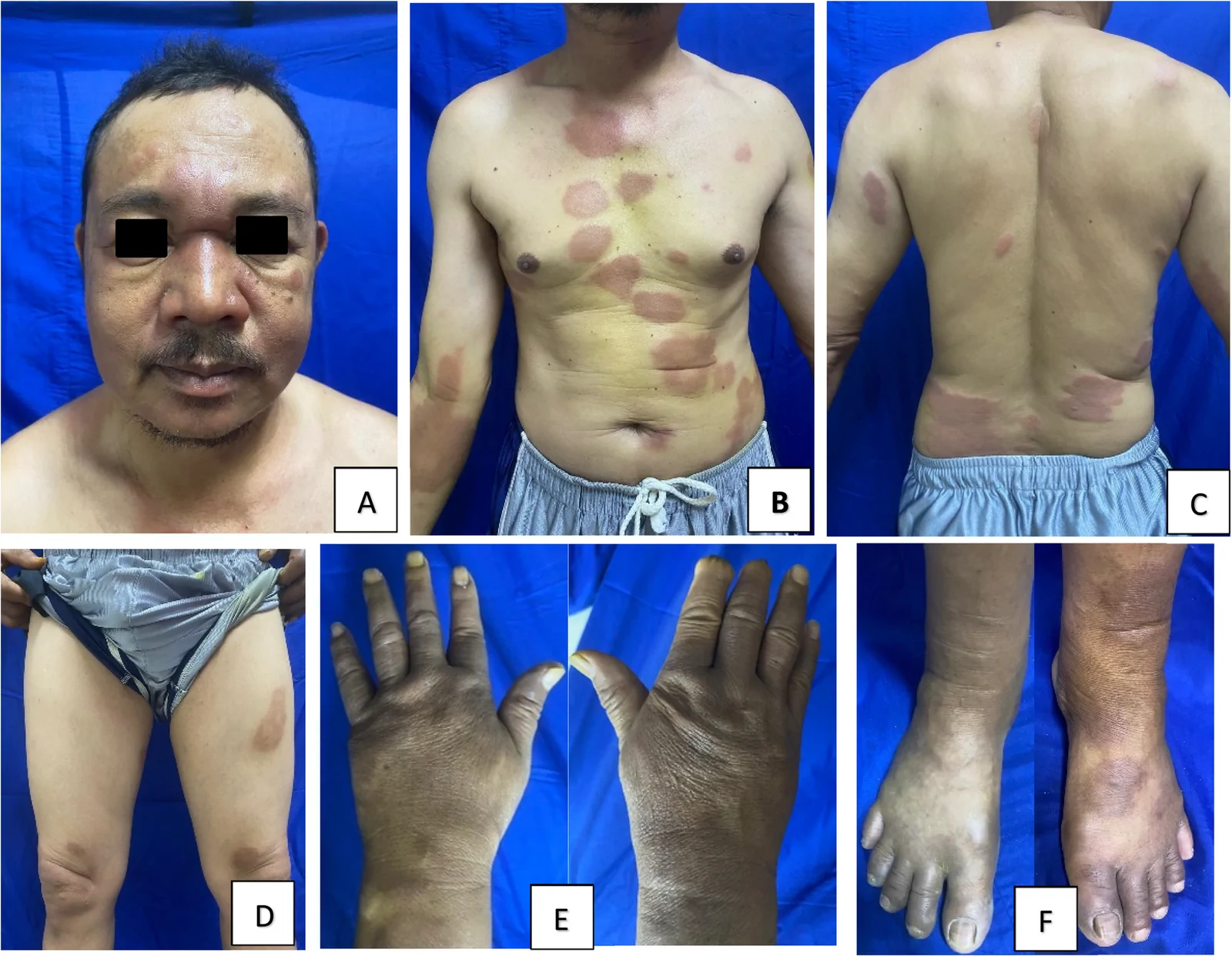
Case 2. Prior to treatment, a physical examination on the first day of observation showed erythematous plaques on the face (**A**), chest and stomach (**B**), back (**C**), and both legs (**D**). Edema in both hands (**E**) and feet (**F**) also observed

**Fig. 5:**
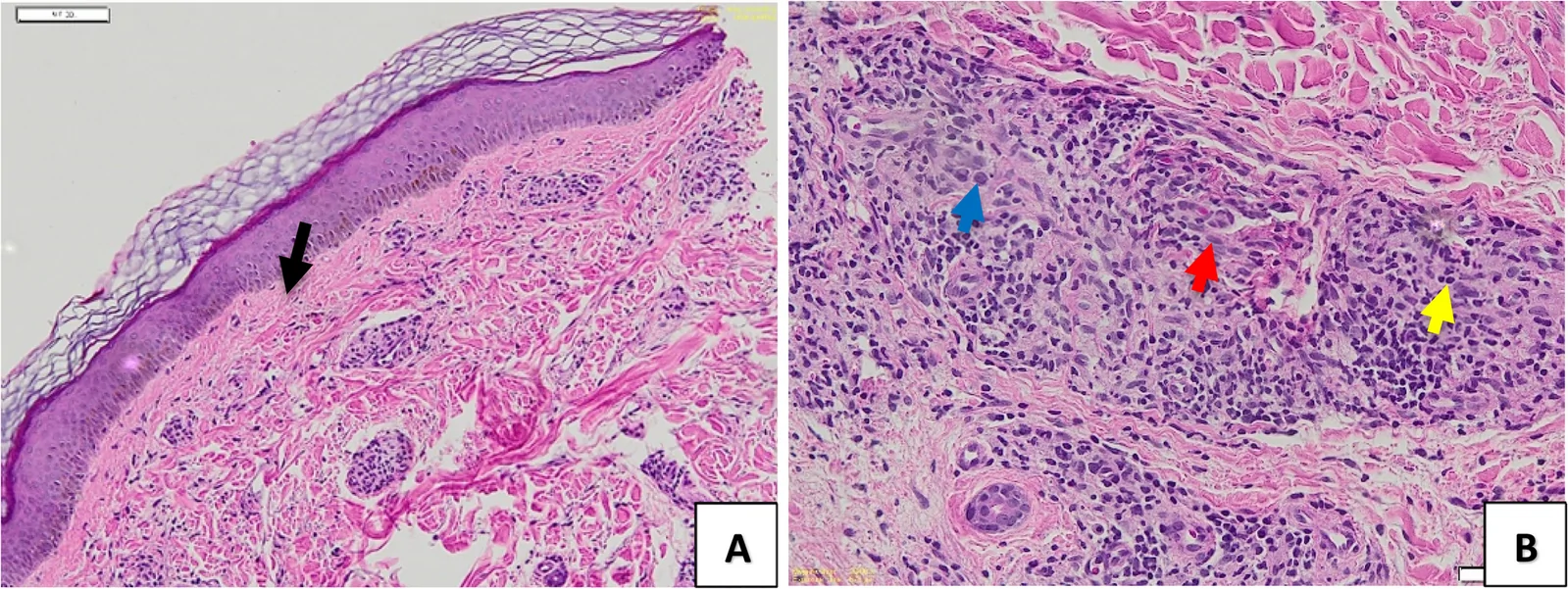
Case 1. The histopathological examination revealed granulomas with a grenz zone (black arrow) supporting the diagnosis of BL-type leprosy (**A**). Inflammatory cells composed of epithelioid cells (red arrow), lymphocytes (yellow arrow), and histiocytes/macrophages (blue arrow) (**B**) (Hematoxylin-eosin stain; original magnification, 100x (**A**), 200x (**B**), respectively)

**Fig. 6:**
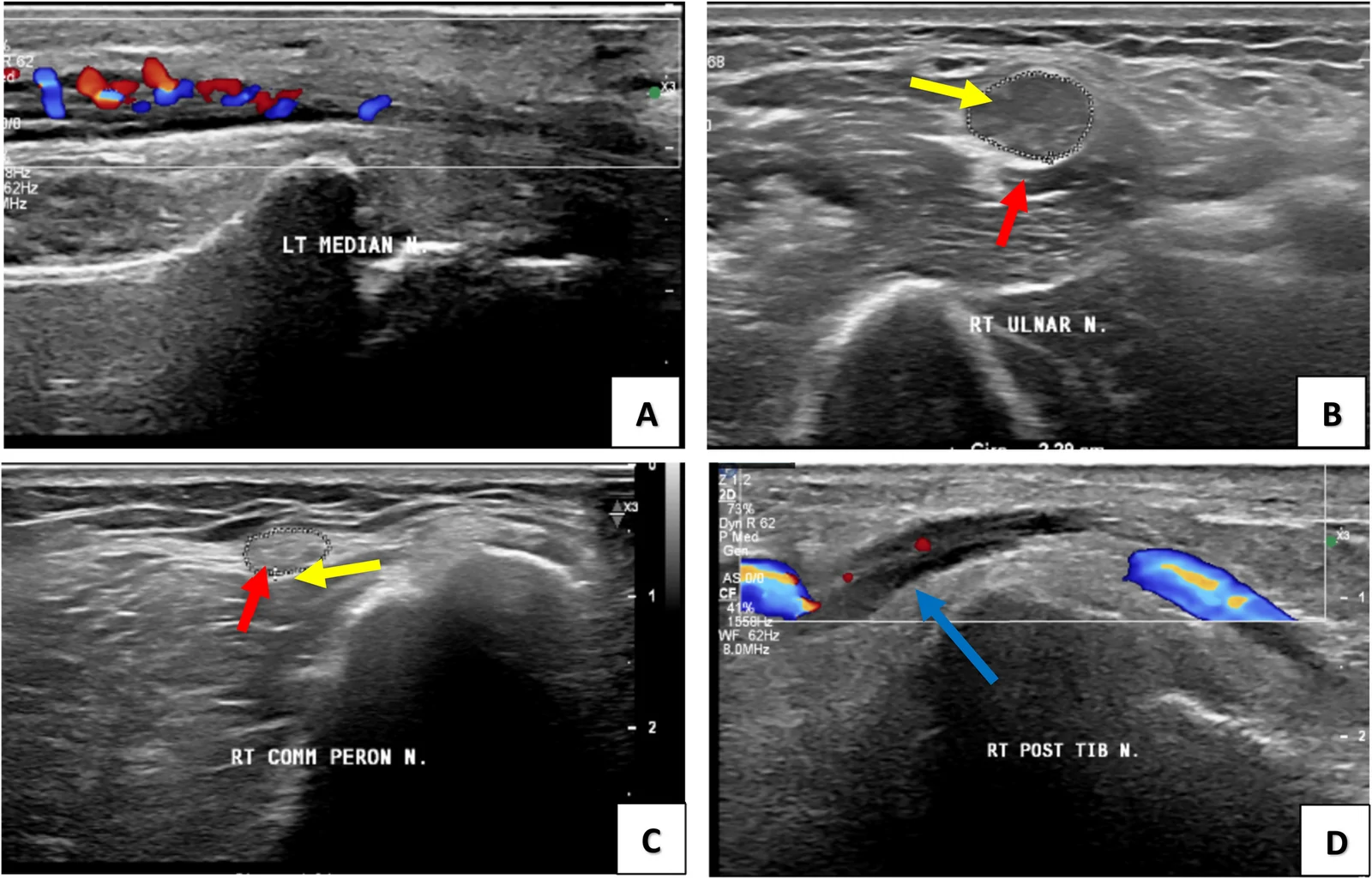
Case 2. Pre-treatment, HRUSG of the peripheral nerve in BL-type leprosy revealed that the median (**A**), ulnar (**B**), common peroneal (**C**), and posterior tibial nerves (**D**) on both sides were hypoechoic (yellow arrow), increase CSA (red arrow), and showed hypervascularity (blue arrow)

**Fig. 7:**
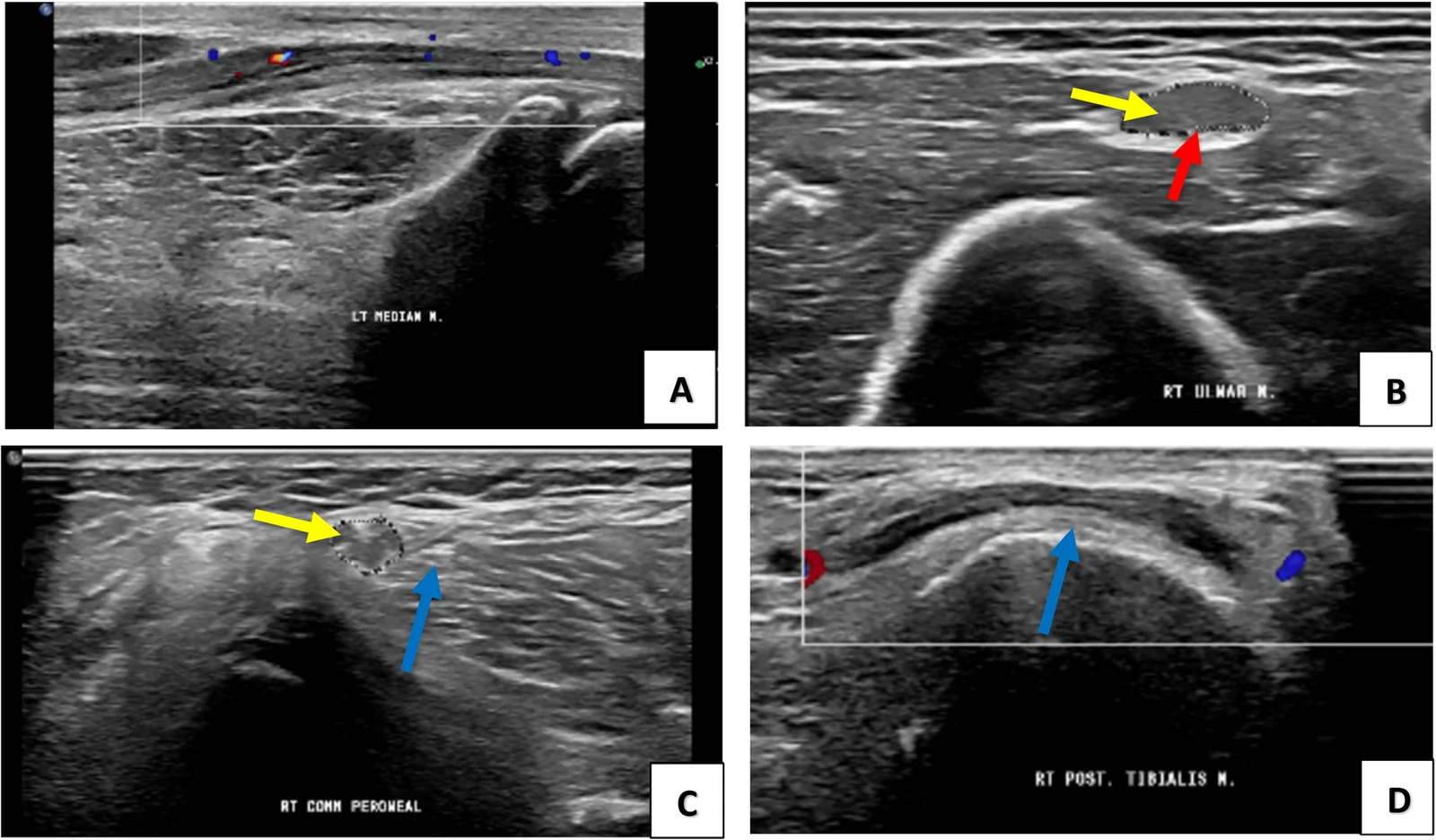
Case 2. Post-treatment, HRUSG of the patient's peripheral nerves revealed that their condition had improved. A significant decrease in CSA (red arrow) was observed along the ulnar (**A**), median (**B**), common peroneal (**C**), and posterior tibial nerves (**D**), and there was no hypervascularity (blue arrow) in the common peroneal (**C**) and posterior tibial nerves (**D**). However, hypoechogenicity persisted (yellow arrow)

A 47-year-old male from an endemic area presented with erythematous plaques on the face, chest, stomach, back, both arms, and legs, which were neither pruritic nor painful (Fig. 4A − D). Approximately five months before consulting, there was a reddish macule on the stomach that did not feel itchy or painful. Four months later, the patient’s symptoms increased in number and expanded to the face, chest, back, both arms, and legs. Two weeks prior to consulting, the patient noticed a gradual increase in the redness of the skin lesions, including the face, which were accompanied by painful and swollen hands and feet (Fig. 4E and F), without fever. Prior to the past two weeks, the patient had never experienced similar symptoms. The plaques showed decreased sensitivity on the stomach and both arms, while the right thigh displayed anesthesia. Furthermore, the presence of stocking anesthesia was detected. Peripheral nerve enlargement was not observed. No motor weakness was seen; however, motor reflexes were not assessed. Slit-skin smear revealed a bacterial index of 3.8 + and morphological index of 90.54%. Histopathology confirmed BL leprosy with perineural fibrosis (Fig. 5A and B).PCR examination was also not done. The patient received MDT-multibacillary (MB) for 12 months and 40 mg of prednisone daily, with tapering conducted biweekly. HRUSG prior to the administration of MDT with the same tool and method revealed hypoechogenicity, increased CSA, and hypervascularity in multiple nerves, despite absence of palpable thickening (Fig. 6A − D). After 2 months of MDT and corticosteroids, CSA and hypervascularity decreased, while hypoechogenicity persisted (Fig. 7A − D). The results of the HRUSG examination are presented in Table 1.

**Table 1:** Peripheral Nerve HRUSG Findings Before and After MDT

Nerve	Case	CSA Before MDT (mm²)	CSA After MDT (mm²)	Vascularity Before MDT	Vascularity After MDT
Median (R)	TT	9.9	8.1	-	-
Median (L)	TT	8.7	8.0	-	-
Ulnar (R)	TT	7.5	7.2	-	-
Ulnar (L)	TT	5.4	6.1	-	-
Common peroneal (R)	TT	7.5	8.0	-	-
Common peroneal (L)	TT	6.9	7.4	-	-
Posterior tibial (R)	TT	5.8	5.4	-	-
Posterior tibial (L)	TT	6.3	6.9	-	-
Median (R)	BL	20.2	17.9	+	+
Median (L)	BL	21.9	20.3	+	+
Ulnar (R)	BL	37.0	27.2	+	+
Ulnar (L)	BL	19.7	16.7	+	+
Common peroneal (R)	BL	17.2	9.8	+	-
Common peroneal (L)	BL	16.9	15.0	+	-
Posterior tibial (R)	BL	15.9	13.5	+	-
Posterior tibial (L)	BL	11.3	8.5	+	-

Peripheral nerve HRUSG findings in two leprosy patients pre- and post-treatment. CSA values are expressed in mm². Echogenicity is described as normal or hypoechoic. Hypervascularity was qualitatively assessed using CD and categorized as present or absent

## Discussion

Leprosy is the most prevalent curable infectious disease affecting the peripheral nervous system [8]. Despite the number of new cases reported globally having decreased from 228,474 in 2010 to 172,711 in 2024, there has been no change in the prevalence of grade 2 disabilities [2, 8]. Therefore, early diagnosis and treatment of nerve impairment in leprosy is important in order to prevent disability [8].

Early detection of leprosy neuropathy is crucial for preventing peripheral nerve impairment. The nerves that are predominantly affected include the ulnar, median, common peroneal, and posterior tibial nerves [9]. Normally, assessment of nerve involvement is done clinically by nerve palpation [6]. Assessment of peripheral nerve involvement through palpation is subjective and may vary among clinicians [10]. Moreover, this examination is a low-accuracy method because it is difficult to recognize nerve thickness until it becomes markedly apparent, particularly when there are no other clinical lesions of leprosy or sensory loss present [11]. There are more objective methods, such as HRUSG, to measure nerve thickening [10].

High-resolution USG is a non-invasive method that provides objective, real-time information and closely reflects the pathological mechanisms present in the nerve [12]. This examination utilizes a high-frequency broadband linear array transducer, ranging between 12 and 15 megahertz [13]. HRUSG can aid in identifying nerve thickenings before clinical palpation. In addition, CD enhances the visualization of vascular channels and blood flow signals within the nerve [14, 15]. Therefore, HRUSG has the potential to become the primary modality for the assessment of peripheral nerves due to its accessibility and lack of contraindications [11]. The assessment of peripheral nerve ultrasound examination consists of echogenicity, CSA, and vascularity. Any abnormality of these parameters indicates nerve involvement [12]. This case report revealed that neither case 1 nor case 2 displayed nerve enlargement upon palpation, prompting an ultrasound examination to assess peripheral nerve involvement both pre- and post-treatment.

Echogenicity refers to the ability of neural tissue to reflect sound waves during HRUSG examinations. There are three classifications of tissue echogenicity: (1) hypoechoic, characterized by a darker appearance compared to surrounding tissue; (2) isoechoic, where the tissue exhibits a similar appearance to adjacent tissue; and (3) hyperechoic, indicated by a lighter appearance relative to surrounding tissue. The echogenicity of tissue serves as a parameter for assessing potential damage to its structure [16]. The echogenicity of the nerves can be graded according to the following criteria: mild = some hypoechogenicity; moderate = obvious hypoechogenicity; severe = absence of any fascicular pattern [8]. Similar to other inflammatory neuropathies, hypoechogenicity may reflect intraneural edema and active inflammation, whereas hyperechogenicity may indicate chronic changes such as fibrosis and structural nerve damage [17]. 

The CSA of the nerve is obtained from the area enclosed by the inner boundary of the hyperechoic rim at the level of maximum thickening [18]. Jain et al. [5] documented that the typical CSA of the median nerve is 6.2 ± 2.2 mm², the ulnar nerve 8.5 ± 3.5 mm², the common peroneal nerve 5.9 ± 3.2 mm², and the posterior tibial nerve 6.3 ± 3.2 mm². Nerve thickening is characterized as an increase CSA of 1 mm² or greater [15]. HRUSG also found hypervascularity in signals of blood flow in the perineural plexus or intrafascicular vessels that are considered a marker of active nerve inflammation in leprosy [12]. Nevertheless, interpretation predominantly remains qualitative due to the absence of standardized quantitative criteria or formal grading methods for vascularity evaluation in the literature. In this case report, the TT-type leprosy patient’s HRUSG examination before treatment showed normal nerve appearance. On the other hand, the BL-type leprosy patient’s nerves had reduced echogenicity, higher CSA, and more hypervascularity (Table 1).

Sreejith et al. [10] reported that 31 out of 70 nerves showed thickening on a clinical examination, while 50 out of 70 nerves showed thickening on an HRUSG, which was statistically significant. The other study indicates that HRUSG had better sensitivity compared to clinical testing in identifying peripheral nerve thickness in leprosy cases [10, 11]. In this case report, the patient with BL-type leprosy showed differences between the nerve examination results obtained through palpation and HRUSG. Palpation revealed no evidence of nerve enlargement, whereas HRUSG examination demonstrated decreased echogenicity, increased CSA, and hypervascularity. In contrast, patient with TT-type leprosy exhibited no sensory impairment upon physical examination, a finding that was consistent with the absence of nerve abnormalities on HRUSG examination.

Jain et al. [5] conducted a research study that identified enlargement in the nerves associated with sensory loss in 23 out of 30 nerves from 12 individuals. In 15 patients who were exhibiting motor weakness, the HRUSG revealed thickening in 17 nerves out of 26. Among the 16 nerves from nine patients presenting with motor and sensory loss, 13 demonstrated sonographic enlargement. A significant correlation was found between clinical factors such as degree of thickening, loss of sensation, and muscle weakness, as well as HRUSG abnormalities such as CSA, echogenicity, and vascularity. The application of ultrasound in leprosy extends beyond the diagnosis of nerve involvement; it also facilitates the monitoring of progressive nerve damage [4, 5]. Our BL case reflects similar findings, exhibiting persistent hypoechogenicity despite improvements in CSA and vascularity, potentially indicating remaining structural damage.

Lugao et al. [4] reported the majority (77.8%) of MB patients exhibited at least one nerve with an elevated CSA, compared to 40.6% of the PB. Furthermore, the study also demonstrated a higher prevalence of elevated CSA in at least one nerve in patients who exhibited reactions (66.7%) compared to those who did not (38.7%). Jain et al. [5] also reported that patients with type 1 or type 2 reactions, characterized by increased vascularity, more frequently exhibited nerve enlargement in both clinically involved nerves and nerves distant from the site of clinical involvement. In this case report, the BL-type leprosy patient with a severe RR showed a progressive increase CSA in all examined nerves before MDT, while the TT-type leprosy patient without a leprosy reaction showed no such increase.

In accordance with the World Health Organization guidelines, a six-month course of MDT-PB treatment was administered for TT-type leprosy. The regimen consisted of rifampicin 600 mg/month and dapsone 100 mg/day. A 12-month course of MDT-MB was prescribed for BL-type leprosy, comprising rifampicin 600 mg/month, dapsone 100 mg/month, and clofazimine 300 mg/month, with a daily dose of 50 mg thereafter [19]. Tambe et al. [20] conducted a comparative analysis of CSA pre- and post-treatment and oral steroid treatment on both the right and left sides of all nerves. The findings indicated a statistically significant difference pre- and post-treatment and oral steroid treatment. Various studies also suggest that HRUSG could serve as an objective and repeatable method to assess the effectiveness of the treatment [20, 21]. However, nerve thickening may continue even after sufficient MDT and does not inherently signify active inflammation. Longitudinal ultrasonographic studies have shown that structural nerve abnormalities may remain stable or worsen post-treatment, possibly indicating chronic damage such as fibrosis or persistent architectural changes rather than acute neuritis [4]. Additionally, as a management strategy for leprosy reactions, the patient received 40 mg of prednisone per day for two weeks. That dosage gradually tapered off as the patient’s condition improved. Both patients in this case report underwent repeated ultrasound examinations after MDT treatment. The TT patient exhibited no structural or vascular abnormalities at baseline, and nerve parameters remained stable during MDT, while the patient with BL-type leprosy underwent a two-month course of MDT-MB and prednisone, which resulted in a notable improvement in both the patient’s clinical appearance and the results of a HRUSG examination. This examination demonstrated a reduction in the CSA across all nerves (Table 1) and the disappearance of hypervascularity of the common peroneal nerve and posterior tibial nerve, while hypoechogenicity remained. This may indicate residual structural disruption of the fascicular architecture, suggesting that different sonographic parameters reflect distinct pathological processes, some potentially reversible and others representing longer-term structural damage, including enlarged CSA indicative of nerve enlargement, increased blood flow signals in the epineurium and endoneurium suggesting active inflammation, and alterations in echo texture and fascicular architecture reflected as changes in echogenicity [6, 17]. 

These findings highlight the potential of HRUSG as a complementary diagnostic tool and as a marker of structural and vascular changes in leprosy neuropathy, especially in the leprosy recation and subclinical neuropathy. The ability of HRUSG to detect nerve abnormalities in the absence of clinically palpable thickening may have implications for earlier recognition, particularly in endemic settings where delayed diagnosis contributes to disability. However, despite the observed structural improvement on HRUSG, sensory testing and disability grading remained unchanged, suggesting that structural changes may not directly correlate with functional recovery. Therefore, the use of HRUSG as a standalone tool for monitoring therapeutic response should be interpreted with caution. This report is limited by the short follow-up duration, which may not capture long-term nerve changes. Measurements were performed once at each site, which may have introduced variability. Independent validation was not performed, representing an additional limitation of this study. Furthermore, electrophysiological studies were not conducted in parallel, thereby limiting the correlation between structural and functional recovery. Consequently, the extent and nature of nerve damage, including demyelination and axonal degeneration, could not be definitively determined.

## Conclusion

Leprosy neuropathy provides a unique human model of infection-triggered inflammatory neuropathy, in which structural and vascular changes may be visualized using HRUSG. This case report indicates that serial HRUSG findings indicate possible alterations after MDT and corticosteroid treatment; nevertheless, the analysis is constrained by insufficient clinical and neurophysiological correlation. Therefore, HRUSG may function as a potential adjunctive tool; however, its significance in therapy monitoring and early diagnosis must be approached with careful consideration. Further prospective studies with larger cohorts are needed to validate these findings and to establish standardized sonographic criteria for nerve assessment in leprosy.

## Data Availability

No datasets were generated or analysed during the current study.

## Abbreviations

BL: Borderline lepromatous leprosy
CD: Color Doppler
CSA: Cross-sectional area
HRUSG: High-resolution ultrasonography
MB: Multibacillary
MDT: Multi-drug therapy
M. leprae: *Mycobacterium leprae*
PB: Paucibacillary
RR: Reversal reaction
TT: Tuberculoid leprosy
